# Supplementary material for: Grafted Microparticles Based on Glycidyl Methacrylate, Hydroxyethyl Methacrylate and Sodium Hyaluronate: Synthesis, Characterization, Adsorption and Release Studies of Metronidazole
Source: Polymers (Basel). 2022 Oct 3;14(19):4151. doi: 10.3390/polym14194151 (PMC9572090; doi:10.3390/polym14194151)
Supplement: Supplementary file 1 [file polymers-14-04151-s001.zip › polymers-1911648-supplementary.pdf]

polymers

MDPI

# Supplementary Material: Grafted Microparticles Based on Glycidyl Methacrylate, Hydroxyethyl Methacrylate and Sodium Hyaluronate: Synthesis, Characterization, Adsorption and Release Studies of Metronidazole

**Aurica Ionela Gugoasa, Stefania Racovita, Silvia Vasiliu and Marcel Popa**

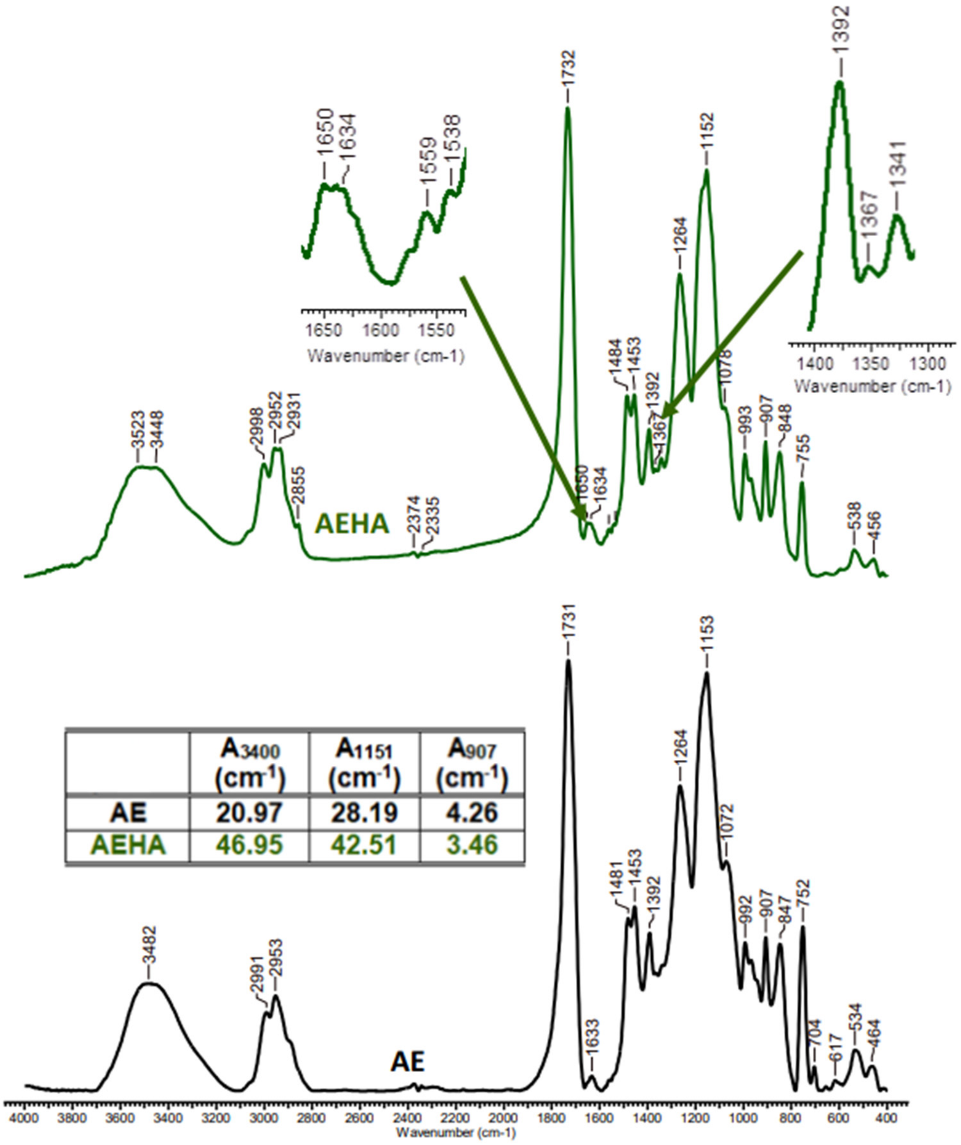


**Figure S1.** The infrared spectra of AE and AEHA microparticles.

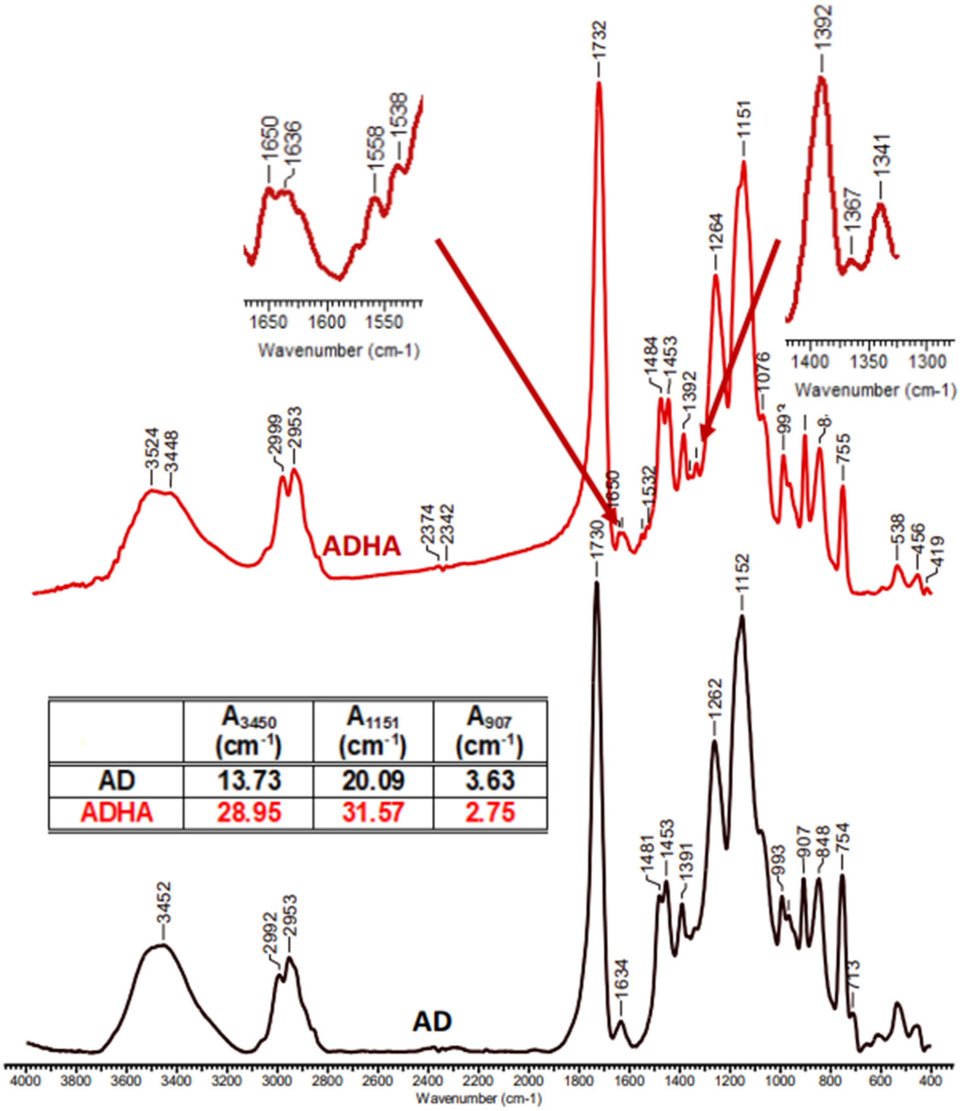


| | $A_{3450}$ ($cm^{-1}$) | $A_{1151}$ ($cm^{-1}$) | $A_{907}$ ($cm^{-1}$) |
|---|---|---|---|
| AD | 13.73 | 20.09 | 3.63 |
| ADHA | 28.95 | 31.57 | 2.75 |

**Figure S2.** The infrared spectra of AD and ADHA microparticles.

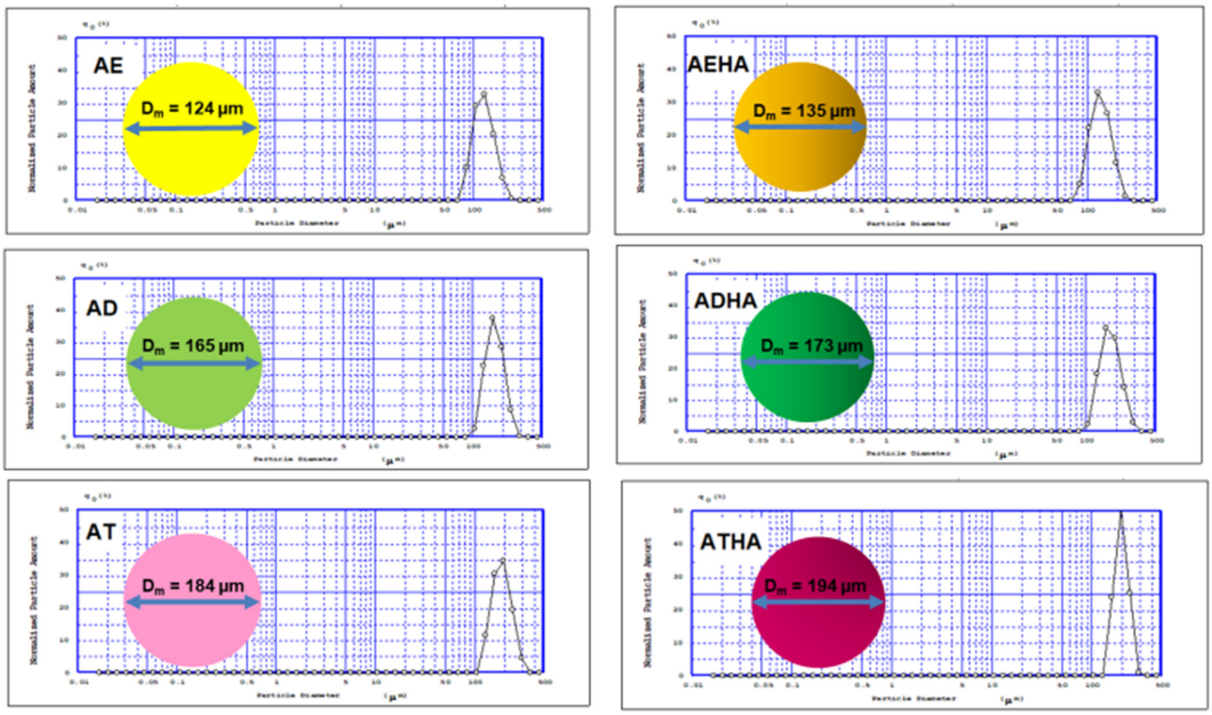


**Figure S3.** Particle size distributions of precursor/hybrid microparticles.
